# The efficacy of insulin degludec and insulin glargine over NPH insulin among toddlers and preschoolers with type 1 diabetes using glycemic variability and time in range

**DOI:** 10.1007/s00431-023-04857-w

**Published:** 2023-02-17

**Authors:** Safinaz Adel Elhabashy, Eman Mohamed Sakr, Nouran Yousef Salah

**Affiliations:** Pediatrics Department, Ain Shams University, Cairo, Egypt

**Keywords:** Insulin glargine, Insulin degludec, NPH insulin, Toddlers, Preschool children, Type 1 diabetes

## Abstract

Optimizing glycemic control without risking hypoglycemia is crucial in toddlers and preschoolers with type 1 diabetes (T1D) to avoid cognitive impairment later in life. Hence, this study aims to compare glycemic parameters among toddlers and preschoolers with T1D in relation to different basal insulins. Sixty toddlers and preschoolers with T1D with mean age of 3.53 ± 1.17 years (range, 2–6) and mean diabetes duration of 9.37 ± 1.85 months were randomly assigned into three equal groups; group A received insulin degludec, group B received insulin glargine, and group C were on NPH. At baseline, the three groups were matched regarding clinical and laboratory parameters (*p* > 0.05). They were followed up at 3 and 6 months for insulin daily dose (IDD), hypoglycemia and severe-hypoglycemia frequency, and glycated hemoglobin (HbA1c). At the study endpoint, continuous glucose monitoring (CGM) was assessed in a random sample of 10 patients from each group. The mean time in range (TIR) of the studied cohort was 55.07 ± 24.05%, and their mean coefficient of variation (CV) was 42.82 ± 11.69%. The TIR was significantly higher in the degludec group (69.36 ± 18.54) and the glargine group (55.43 ± 26.51) than the NPH group (32.56 ± 9.11), *p* < 0.001. Meanwhile, the CV was significantly lower in the degludec group (35.12 ± 6.47) than the gargine (44.1 ± 13.13) and the NPH (53.8 ± 7.54) groups, *p* < 0.001. The insulin degludec and glargine groups had significantly lower HbA1c (*p* = 0.002), hypoglycemia (*p* = 0.006), severe hypoglycemia (*p* = 0.029), and IDD (*p* = 0.015) than the NPH group.

*Conclusion*: Insulin degludec and glargine resulted in better HbA1c and TIR with reduced hypoglycemia and IDD than NPH among toddlers and preschoolers with T1D. Moreover, CV was lowest in the insulin degludec group.

**Table:** 

**What is Known:** *• Insulin therapy is the mainstay of T1D management.* *• Optimal insulin therapy for young children with T1D should provide effective glycemic.*	
**What is New:** *• Insulin degludec and insulin glargine have better efficacy than NPH insulin among toddlers and preschoolers with T1D in the term of significantly lower coefficient of variation, HbA1c and IDD and significantly higher time in range.* *• Insulin degludec and insulin glargine have better safety in the term of less hypoglycemia and severe hypoglycemia episodes than NPH insulin among toddlers and preschoolers with T1D.*

**What is Known:**

*• Insulin therapy is the mainstay of T1D management.*

*• Optimal insulin therapy for young children with T1D should provide effective glycemic.*

**What is New:**

*• Insulin degludec and insulin glargine have better efficacy than NPH insulin among toddlers and preschoolers with T1D in the term of significantly lower coefficient of variation, HbA1c and IDD and significantly higher time in range.*

*• Insulin degludec and insulin glargine have better safety in the term of less hypoglycemia and severe hypoglycemia episodes than NPH insulin among toddlers and preschoolers with T1D.*

## Introduction

The incidence of type 1 diabetes (T1D) in toddlers and preschool children is rising in many parts of the world [1]. Managing T1D in young children presents a combination of challenges to their families and healthcare providers. A major challenge is the difficulty in achieving metabolic control without risking hypoglycemia. This is attributed to the young child’s developmental immaturity, limited communication, cognitive, and emotional maturity and their heterogeneous and often unpredictable lifestyles (variable exercise, eating and sleeping patterns, dependence on caregivers for injections and blood tests) [2].

The low cognitive ability and immature communication among young children with T1D impair their ability to express their ill feeling resulting from hypoglycemia or hyperglycemia. Moreover, nocturnal hypoglycemia is common and represents a major hazard in young children with T1D and can be undiagnosed unless continuous glucose monitoring is used [3].

Hypoglycemia and its detrimental neuropsychological sequela are of far greater concern for toddlers and preschoolers than for older children and adolescents. Even mild hypoglycemia can result in cognitive dysfunction manifested by mental inflexibility and dissociative learning [4].

Recently, the role of glycemic fluctuations in cognitive impairment among toddlers and preschoolers with T1D has emerged [5]. Hence, optimal insulin therapy for young children with T1D should provide effective glycemic control while minimizing the risk of hypoglycemia and hyperglycemia. This requires balancing the risk of long-term complications from wide fluctuations in blood glucose levels and hyperglycemia with the fear of acute hypoglycemia [6].

Although glycated hemoglobin (HbA1c) is the gold standard for assessing glycemic control, such monitoring is unable to reliably measure acute glycemic excursions and glycemic fluctuations. Continuous glucose monitoring (CGM) has been shown to allow a more comprehensive assessment of overall glycemic control together with time in range and glycemic variability [7].

Insulin therapy that mimics normal physiological patterns as closely as possible remains the main goal of treatment for T1D. Ideally, a physiological, flexible, and predictable insulin regimen protecting against hypoglycemia and inappropriate weight gain is needed. Continuous subcutaneous insulin infusion is an appropriate mean of achieving this goal, but may not be affordable or available, and is not suitable for all children. Basal bolus insulin therapy using modern basal and rapid‐acting analogs has the potential to offer a more physiological insulin profile, than conventional human insulins, with improved safety [8].

Insulin degludec is an ultra-long acting basal insulin analog indicated for T1D and type 2 diabetes (T2D) in children as young as 1 year of age [9]. It has a unique mode of action that provides an ultra-long duration of action, with low day-to-day variability in blood glucose level compared with other basal insulins [10]. Randomized controlled trials (RCTs) in adults have demonstrated that insulin degludec is associated with a reduced risk of hypoglycemia than other insulin analogs, at equivalent levels of glycemic control [11–13].

Few clinical trials investigated the use of different basal insulins in children and adolescents with T1D in daily life. Moreover, data from clinical trials in adults with T1D are not readily transferable to the pediatric patients of different age groups. Studies have shown the different pharmacokinetic profile of insulin analogs in young children and adolescents compared to adults [14]. Hence, there is an unmet need to study the effects of different basal insulins in each age group separately [15].

## Aim

The primary endpoint of the study was to assess the efficacy in terms of HbA1c and safety in terms of frequency of hypoglycemic episodes among toddlers and preschool children with T1D on insulin degludec, insulin glargine, and NPH; while the secondary end point was to assess the glycemic variability and time in range at the study endpoint.

## Methodology

This prospective randomized, three-armed trial was approved by the Ethical Committee of Ain Shams University, and an informed consent was obtained from each legal guardian before participation. Reporting of the study conforms to the Consolidated Standards of Reporting Trials 2010 statement [16]. The study was registered in the ClinicalTrials.gov (NCT04664764). Sixty children with T1D were recruited from the regular attendees of the Pediatric and Adolescents Diabetology Unit (PADU), Children’s Hospital, Ain Shams University. In Egypt, access to health services and medication is assured by health insurance law. The cost of degludec and insulin glargine far exceeds that of NPH insulin which is still available for use in the Ministry of health care facilities, and also in PADU, Ain Shams University.

By extensively reviewing the literature, no previous studies were found to compare the effectiveness of inulin degludec to both insulin glargine and NPH insulin in type 1 diabetic children in terms of glycosylated hemoglobin (HbA1c) level and frequency of hypoglycemia episodes. So, a sample size of 60 patients with type 1 diabetes mellitus was recommended to be divided into 3 groups (20 patients per group) according to the basal insulin used based on experts’ recommendation in the field of pediatrics “pilot study.”

Patients were defined according to the criteria of the ISPAD 2018 [17]. Patients with T1D on regular insulin therapy, aged between 2 and 6 years, diagnosed for at least 6 months were included. Exclusion criteria included patients with associated medical conditions such as celiac disease or autoimmune thyroiditis, patients with other types of diabetes mellitus, patients with history of liver disease, or any disorder likely to impair liver functions or elevated liver enzymes, renal impairment due to causes other than diabetes, presence of hypertension, patients on any vitamins, or food supplements 1 month before study and participation in a previous investigational drug study within 3 months preceding screening. All participants were asked to refrain from substantial changes in their lifestyle habits in the course of the study. By extensive reviewing of the literature, no previous studies were found to compare the efficacy and safety of insulin degludec to both insulin glargine and NPH insulin in young with T1D in terms of HbA1c level and frequency of hypoglycemic episodes.

### Randomization and study groups

Eligible children were assigned by simple randomization. Those patients less than 6 years, who showed up in the outpatient diabetology clinic, were collected over a period of 3 months. Prior to the study, the children were on NPH insulin and insulin glargine. At baseline, all participants were randomly assigned to one of the treatment groups and they were put under regular follow-up weekly for 1 month to adjust the basal dose and for education. Accordingly, group A were those who received insulin degludec (Tresiba; Novo Nordisk, Bagsvaerd, Denmark) once per day, group B were on insulin glargine once per day (Lantus®; Sanofi-Aventis, Frankfurt, Germany), and group C on NPH insulin (NPH, human isophane insulin®; Novo Nordisk A/S) twice daily. The 3 groups received insulin Aspart (NovoRapid®, Novo Nordisk, Copenhagen, Denmark) as the mealtime insulin, three times daily.

During the treatment period, parents were asked to measure SMBG 7 times daily, and gluco-strips were made available for them for the whole study period. The basal-bolus insulin doses were adjusted according to ISPAD guidelines [15] aiming for a fasting/pre-prandial SMBG target of 70–120 mg/dL and a postprandial of 90–198 mg/dL. The patients were followed monthly during the study period. They were exposed to similar educational gains all through.

## Baseline clinical assessment

All included toddlers and young children with T1D were subjected to baseline detailed medical history taking with special stress on demographic data, age of onset of diabetes, disease duration, history of acute complications, i.e., frequency of hypoglycemia and number of hospital admission by diabetic ketoacidosis (DKA), and history of chronic micro and macro-vascular complications.

Thorough clinical examination was done laying stress on anthropometric measures including weight in kilograms (kg), height in centimeters (cm), and body mass index (BMI) which were plotted against standard deviation scores for age and gender World Health Organization (WHO) [18].

### Sample collection and laboratory analysis

Peripheral blood samples were collected at the start of the study (day 0), at 3 months, and at 6 months on potassium-ethylene diamine tetra-acetic acid (K2-EDTA) in sterile vacutainer tubes (final concentration of 1.5 mg/mL) (Beckton Dickinson, Franklin Lakes, NJ, USA) for assessment of HbA1c [19].

## Patient education

Patients were counseled to exclude taking any vitamins or food supplements a month before study entry and also, and to keep on a fixed diet regimen throughout the study discussing exchange tables, and carb counts, levels and hypoglycemia manifestations, and hyperglycemia and target levels. Patients were taught to check and record their SMBG 7 times daily, and as indicated.

### Follow-up and endpoints

The primary endpoint of this trial was the level of HbA1c and frequency of hypoglycemic episodes after 6 months. All patients were closely and clinically followed up in the diabetology outpatient clinic by 2 of the researchers every 4 weeks during the study period with an assessment of SMBG and frequency of hypoglycemia per patient. Nocturnal plasma glucose values were measured at 03: 00 h as part of the 7‐point SMPG profile. Hypoglycemic episodes were classified according to ISPAD 2018 guidelines; clinical hypoglycemia alert was defined as a glucose value of ≤ 70 mg/dL); serious hypoglycemia as a glucose value of 54 mg/dL. Severe hypoglycemia was defined as an event associated with severe cognitive impairment (including coma and convulsions) requiring external assistance by another person to actively administer carbohydrates, glucagon, or take other corrective actions [20].

### Continuous glucose monitoring (CGM)

At the study endpoint (after 6 months), CGM was applied for a random sample of 10 patients from each group for a period of 5 days each (total of 30 patients). Medtronic iPro2 Recorder CGM system was used through the insertion of a glucose oxidase-based sensor in the subcutaneous area of the abdomen. The system was recording interstitial glucose over 5 days continuously. All candidates were instructed to follow their usual diet and insulin regimen. Calibration of the sensor with the glucometer was done three times daily, to assure accuracy. The recorded data were obtained and downloaded using Medtronic Diabetes, Care Link software. Maximum CGM reading, minimum CGM reading, coefficient of variation (CV) and time in range (TIR) were obtained for each participant. TIR was defined as time spent between 70 and180 mg/dl (3.9–10.0 mmol/L), hyperglycemia was defined as CGM reading > 180 mg/dl (10 mmol/L); while hypoglycemia was defined as CGM reading < 70 mg/dl (3.9 mmol/L) [21].

## Statistical analysis

Data were collected, revised, coded, and entered to the Statistical Package for Social Science (IBM SPSS) version 23. The Kolmogorov–Smirnov test was used to examine the normal distribution of variables. The quantitative data were presented as mean and standard deviations when their distribution was parametric while non-parametric data were presented as the median and interquartile range (IQR). Qualitative data were presented as number and percentages. The comparison between the threegroups as regards qualitative data was done using the chi-square test. The comparison between two independent groups with quantitative data and parametric distribution was done using independent *t*-test while data with non-parametric distribution was analyzed using Mann-Whitey test. To identify within-group changes (before and after 12 weeks of intervention), we applied paired sample *t*-tests for quantitative parametric data and Willcoxon test for quantitative data with non-parametric distribution. Analysis of covariance (ANCOVA) was performed to compare mean values between groups adjusted for differences in baseline measures of IDD and HbA1C. The confidence interval was set to 95% and the margin of error accepted was set to 5%. So, a *p*-value < 0.05 was considered significant.

## Results

### Baseline clinical and laboratory characteristics

Of the sixty young children with T1D enrolled, 27 were males, and the mean age of the group whole was 3.53 ± 1.17 years (range, 2–6). Their mean diabetes duration was 9.37 ± 1.85 months, range 5–12 months. Initially, insulin daily dose (IDD) ranged between 0.35 and 1.5 U/kg/day with mean ± SD 0.90 ± 0.23 units. Their mean body mass index was 18.71 ± 3.23 kg/m2. Patients checked their blood glucose 7 times daily, only one patient checked it twice a day and three patients checked more than 7. The three groups were followed up for 6 months with an assessment of insulin daily dose (IDD), frequency of hypoglycemia and severe hypoglycemia/week, and HbA1c. No significant difference was found between baseline clinical and laboratory data among the three groups (*p* > 0.05), Table 1. The pre-trial regimen of all participants comprised basal-bolus therapy. No serious adverse events were recorded in the three study groups throughout the 6 months of therapy.

**Table 1:** Comparison between toddlers and preschoolers with T1D using different basal insulin at baseline and after 6 months

Variable		**Degludec group (*n*** **= 20)**				**Glargine group (*n*** **= 20)**				**NPH group (*n*** **= 20)**				*P*^2^ value	*P*^1^ value	
		***P*-value**	**Change**	**6 months**	**Baseline**	***P*-value**	**Change**	**6 months**	**Baseline**	***P*-value**	**Change**	**6 months**	**Baseline**			
**Males**	**Gender**	-	-	-	7 (35.0%)	-	-	-	8 (40.0%)	-	-	-	12 (60%)	-	0.243	-
**Age (years)**		-	-	-	3.51 ± 1.17				3.41 ± 1.09	-	-	-	3.69 ± 1.27	-	0.752	-
**Disease duration (months)**		-	-	-	9.36 ± 1.95	-	-	-	9 ± 2.24	-	-	-	9.67 ± 1.5	-	0.243•	-
					7–12				5–11				8–12			
**Weight z score**		0.601	−0.02	−0.33	−0.18	0.232	−0.26	−0.6	−0.38	0.823	−0.01	0.18	0.41		0.207	0.344
			−0.4–0.75	−1.05–1.3	−1.69–0.32		−0.45–0.19	−1.46–0.01	−1.19–0.6		−0.34–0.36	−0.33–0.84	−0.49–1.1			
**Height z score**		0.681	−0.03	−2.32	−2.25	0.014	−0.6	−2.16	−2.11	0.247	−0.27	−1.88	−0.78		0.113	**0.036**
			−0.5–0.43	−5.45 to −0.16	−4.46 to −1.36		−0.76 to −0.28	−3.82 to −0.81	−3.98 to −0.37		−0.5–0.19	−2.74 to −0.77	−3.62–0.02			
**BMI z score**		0.232	0.03	2.25	1.89	0.001	0.73	2.11	2.05	0.36	0.02	2.08	1.41	**-**	0.573	**0.002**
			−0.02–0.35	1.23–3.17	1.04–2.99		0.06–1.87	−0.17–3.19	0.56–2.29		−0.08–0.14	0.85–2.55	0.66–2.29			
**IDD (U/Kg/day)**		0.229	−0.5	0.83 ± 0.20	0.87 ± 0.22	0.032	−0.1	0.79 ± 0.24	0.87 ± 0.27	0.497	0.1	0.99 ± 0.22	0.96 ± 0.21	**0.015**	0.419	0.203
			−0.15–0.08				−0.2–0.03				−0.18–0.2					
**Hypoglycemia frequency/week**		0.033	−2	7	-	0.075	−3	11	-	0.764	1	18.5	-	**0.006**	**-**	**0.014**
			−5–0	5.5–12			−4.5–1	6–15			−2.5–7.5	8–25				
**Severe hypoglycemia frequency/week**		0.017	−3.0	1	-	0.271	−2	2	-	0.491	0.5	5	-	**0.029**	**-**	**0.046**
			−6 to −1	0–2.5			−3–3	0–5			−3–1	1–7				
**Hospital admission/6 months**		-	-	1.35 ± 0.75	-	-	-	1.05 ± 0.83	-	-	-	1.75 ± 0.91	-	**0.034**	**-**	**-**
				1–4				0–3				1–3				
**Average SMBG (mg/dl)**		-	188.5	-	-	-	258.75	-	-	-	261.75	-	-	** < 0.001**	**-**	**-**
			178.75–209				236.5–290.75				226.5–292.75					
**HbA1c (%)**		0.001	−1.55	6.57 ± 1.77	8.12 ± 1.74	0.024	−0.5	7.51 ± 1.88	8.18 ± 2.06	0.09	−0.25	8.52 ± 1.11	9.25 ± 1.77	**0.002**	0.106	**0.034**
			−2.8 to −0.50				−1.2 to −0.15				−2–0.7					

*IDD* insulin daily dose, *SMBG* self monitoring of blood glucose, *HbA1c* fraction C of glycated hemoglobin

*P* value was obtained using analysis of covariance (ANCOVA) unless specified, *: independent t-test was used for data expressed as mean and standard deviation, †: chi-square test was applied for the comparison of data expressed as numbers (percentage), *P*^3^ value was obtained from paired-samples *t*-tests for parametric variables or Wilcoxon rank-sum test for non-parametric variables

*P* comparison within each group, *P*^1^ comparison of the baseline data between the 3 groups, *P*^2^ comparison between the 3 groups at 6 months, *P*^3^ comparison of the data change between the 3 groups

### Endpoint clinical and laboratory characteristics of the study population

Comparison between the three study groups at the end of the study revealed significantly lower HbA1c (*p* = 0.002), frequency of hypoglycemia (*p* = 0.006), and severe hypoglycemia (*p* = 0.029) in the insulin degludec and insulin glargine groups compared to the NPH group. Regarding SMBG readings, average SMBG was significantly lower in the insulin degludec than the insulin glargine (*p* < 0.001) and NPH insulin (*p* < 0.001) groups. Moreover, children on NPH insulin required higher IDD than the insulin degludec (*p* = 0.028) and the insulin glargine groups (*p* = 0.006) and had significantly higher hospital admissions than the insulin glargine group (*p* = 0.01) (Table 2).

**Table 2:** Comparison between toddlers and preschoolers with T1D using different basal insulin after 6 months

**Post Hoc analysis by LSD**			
	**P1**	**P2**	**P3**
IDD (U/kg/day)	0.546	**0.028**	**0.006**
Average SMBG (mg/dl)	** < 0.001**	** < 0.001**	**0.745**
HbA1c (%)	0.070	** < 0.001**	0.055
Hypoglycemia frequency/week	0.335	**0.003**	**0.024**
Severe hypoglycemia frequency /week	0.189	**0.011**	0.131
Frequency of hospital admission/6 months	0.258	0.133	**0.010**

*IDD* insulin daily dose, *SMBG* self monitoring of blood glucose, *HbA1c* fraction C of glycated hemoglobin, *P*-value < 0.05: Significant, •: one-way ANOVA test, ‡: Kruskal Wallis test

P1 degludec vs glargine, P2 degludec vs NPH, P3 glargine vs NPH

### CGM data

**Fig. 1:**
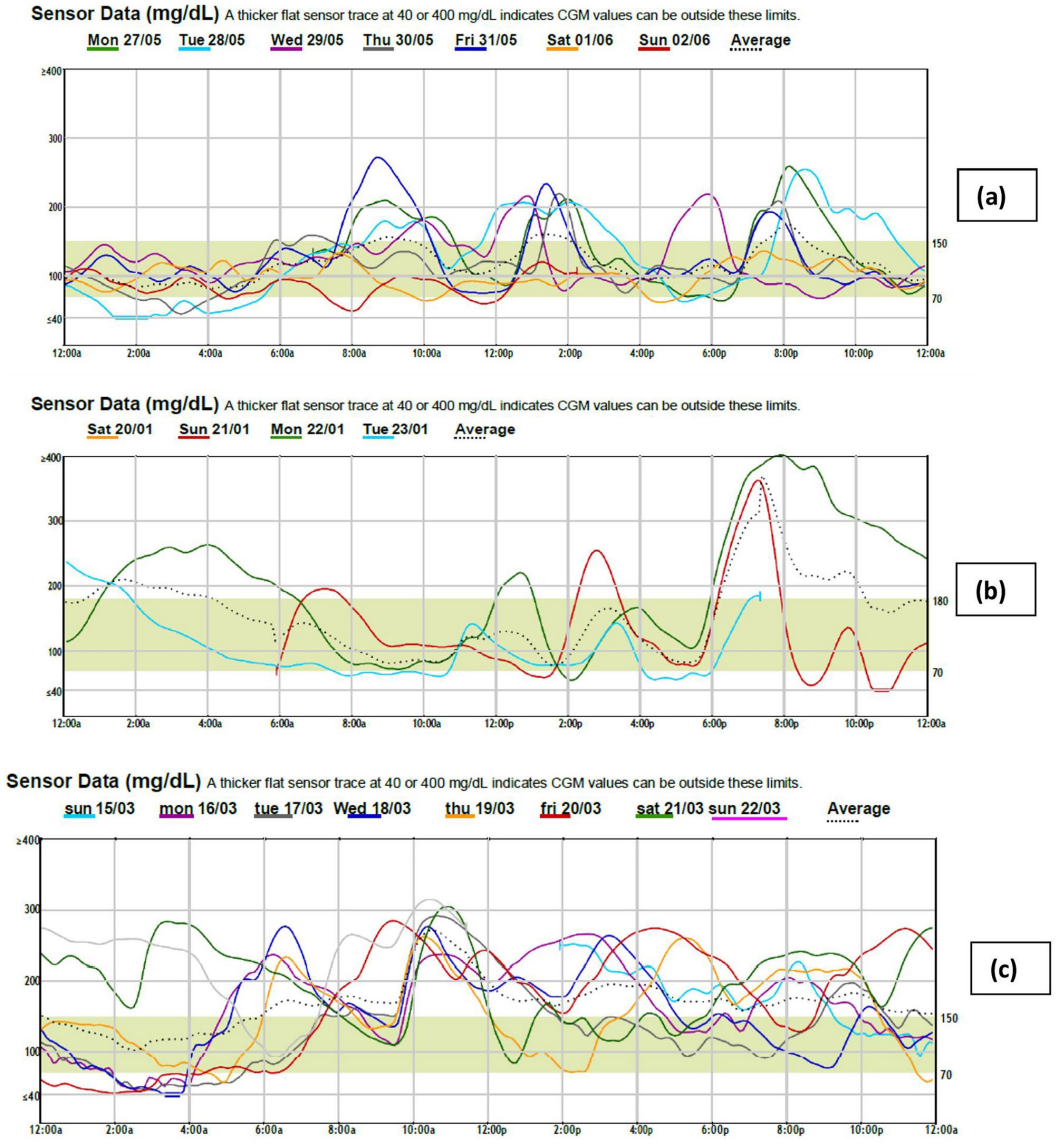
CGM overlay of **a** female patient number 27 aged 2 years 6 months on insulin degludec, **b** male patient number 32 aged 2 years 9 months on insulin glargine, and **c** female patient number 38 aged 3 years on NPH insulin

At the study endpoint, the mean time in the range of the whole studied toddlers and preschoolers with T1D was 55.07 ± 24.05%, range 0–86 and their mean coefficient of variation was 42.82 ± 11.69%, range 23.5–67. The coefficient of variation was lowest in the insulin degludec group, being significantly lower than the other two groups (*p* < 0.001). Time in range was significantly higher in the insulin degludec and the insulin glargine groups than the NPH group (*p* < 0.001) (Fig. 1 and Table 3).

**Table 3:** Comparison of CGM data of toddlers and preschoolers with T1D using different basal insulin after 6 months

		**Type of basal insulin**			**Test value**	***P*-value**
		**Degludec group (*n* = 10)**	**Glargine group (*n* = 10)**	**NPH group (*n* = 10)**		
Average CGM reading (mg/dl)	Mean ± SD	193 ± 18.77	242.17 ± 22.19	276.79 ± 28.31	36.062•	** < 0.001**
	Range	162.5–236.5	222.5–277.5	241.5–319		
Time in range (%)	Mean ± SD	69.36 ± 18.54	55.43 ± 26.51	32.56 ± 9.11	10.718•	** < 0.001**
	Range	29–86	0–76	22–47		
Time in hypoglycemia (%)	Median (IQR)	5 (3–8)	5 (3–9)	11 (8–11)	3.387‡	0.184
	Range	0–14	0–9	2–15		
Time in hyperglycemia (%)	Median (IQR)	18 (14–25)	33 (19–43)	58 (57–63)	11.227‡	**0.004**
	Range	13–68	17–100	42–63		
Coefficient of variation (%)	Mean ± SD	35.12 ± 6.47	44.1 ± 13.13	53.8 ± 7.54	12.783•	** < 0.001**
	Range	26.4–47.3	23.5–67	42.5–62		

**Post hoc analysis by LSD**			
**-**	**P1**	**P2**	**P3**
Maximum CGM reading	** < 0.001**	** < 0.001**	0.165
Average CGM reading	** < 0.001**	** < 0.001**	**0.005**
Time in range (%)	0.117	** < 0.001**	**0.022**
Time in hyperglycemia (%)	**0.039**	**0.004**	**0.025**
Glycemic variability (%)	**0.034**	** < 0.001**	**0.035**

*T1D* type 1 diabetes, *CGM* continuous glucose monitoring, *P*-value < 0.05: Significant, •: one-way ANOVA test, ‡: Kruskal–Wallis test, P1 degludec vs glargine, P2 degludec vs NPH, P3 glargine vs NPH

### Glycemic control

**Fig. 2:**
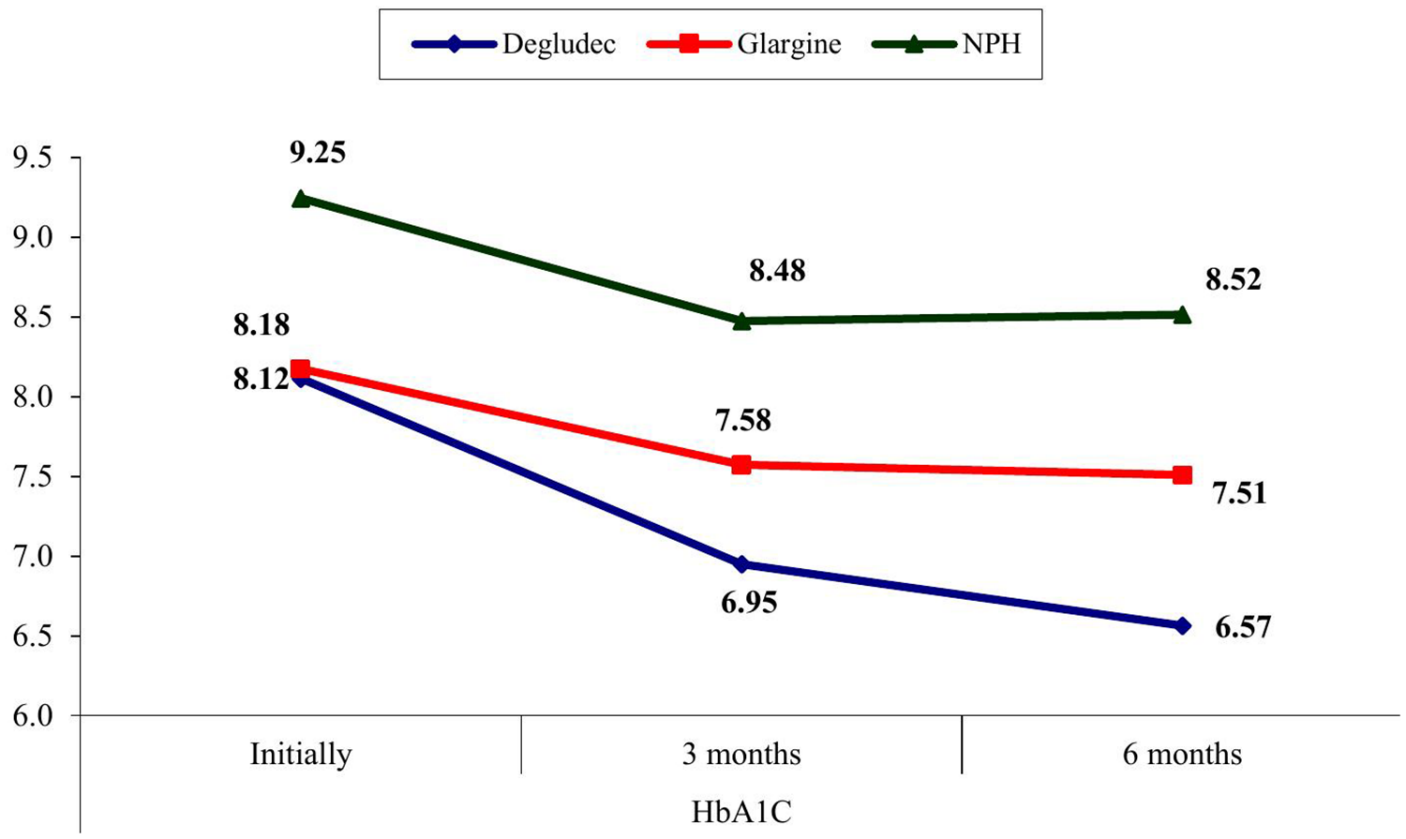
Comparison between toddlers and preschoolers with type 1 diabetes (T1D) receiving insulin degludec, insulin glargine, and NPH insulin regarding HbA1c initially, at 3 months and at 6 months

Comparison between young children with T1D receiving insulin degludec, insulin glargine, and NPH insulin at baseline and at end of the study (at 6 months post-therapy) showed significantly lower HbA1c in the degludec group and the glargine group at the end of the study than baseline (*p* = 0.001 and *p* = 0.024, respectively). On the other side, no significant difference was found between toddlers and preschoolers with T1D who received insulin NPH insulin at baseline and at the end of the study as regards HbA1c (*p* = 0.09) (Fig. 2 and Table 1).

### Hypoglycemic episodes

As regards hypoglycemic episodes, the insulin degludec group showed a significantly lower frequency of hypoglycemia and severe hypoglycemia at the end of the study than baseline (*p* = 0.03 and *p* = 0.017, respectively). However, no significant difference was found in the frequency of hypoglycemia and severe hypoglycemia at the end of the study from baseline in the other two groups (Table 1).

### Insulin dose

**Fig. 3:**
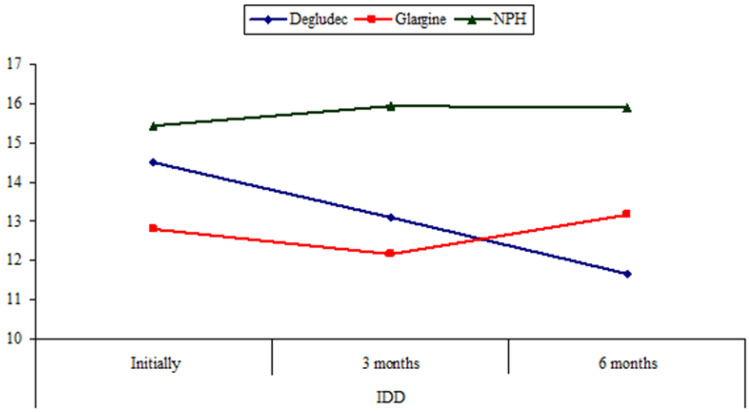
Comparison between toddlers and preschoolers with type 1 diabetes (T1D) receiving insulin glargine, insulin degludec, and NPH insulin as regards insulin daily dose (IDD) U/kg/day initially, at 3 months and at 6 months

The insulin glargine group showed significantly lower IDD at the end of the study than at baseline (*p* = 0.032). While the insulin degludec group and the NPH insulin group showed no significant change in the IDD at the end of the study from baseline (Fig. 3 and Table 1).

Upon comparing toddlers and preschool children with T1D, no significant difference between toddlers and preschool children as regards IDD, HbA1c, and frequency of hypoglycemia and severe hypoglycemia (Table 4).

**Table 4:** Comparison of the clinical and laboratory data among toddlers versus preschool children with T1D at baseline and at 6 months

Variable		Toddlers with T1D (*n* = 28)				Preschool children with T1D (*n* = 32)				*P*^1^-value
		***P*-value**	**Change**	**6 months**	**Baseline**	***P*-value**	**Change**	**6 months**	**Baseline**	
**Female**	**Gender**	–	–	–	7 (43.8%)	–	–	–	26 (59.1%)	0.291
**Males**			–	–	9 (56.3%)		–	–	18 (40.9%)	
**Disease duration (years)**		–	–	–	4.00 ± 1.79	–	–	–	3.28 ± 1.75	0.167
**Weight z score**		0.179	0.19	−0.2	−0.18	0.179	−0.17	−0.3	−0.16(−0.86–0.61)	0.085
			(−0.14–0.95)	(−0.83–1)	(−1.72–0.93)		(−0.42–0.28)	(−0.8–0.72)		
**Height z score**		0.836	−0.16	−1.75	−1.84	0.836	−0.35	−2.01	−1.71	0.587
			(−0.721.19)	(−3.78–-0.67)	(−4.05 to −0.9)		(−0.66–0.13)	(−4.04–-0.62)	(−4.23 to −0.37)	
**BMI z score**		0.059	0.11	1.97	1.57	0.059	0.05	1.89 (0.73–2.55)	1.82 (0.71–2.51)	0.482
			(−0.040.44)	(0.71–2.69)	(0.66–2.25)		(−0.03–0.3)			
**NPH**, *n* (%)	**Type of basal insulin**	–	–	–	5 (31.3%)	–	–	–	15 (34.1%)	0.918
**Degludec**, *n* (%)			–	–	5 (31.3%)		–	–	15 (34.1%)	
**Glargine, *n* (%)**			–	–	6 (37.5%)		–	–	14 (31.8%)	
**IDD (IU/Kg/day)**		0.14	−0.13 (-0.20 – 0.05)	0.89 ± 0.26	0.97 ± 0.30	0.621	−0.05 (0.15–0.10)	0.86 ± 0.23	0.88 ± 0.20	0.134
**Frequency of hypoglycemic attacks/week**		0.36	−0.5 (−3–1)	1 (0–5)	3 (2–4)	0.36	−1(−3–1)	2 (0–5)	4 (2–5)	0.946
**Average SMBG (mg/dl)**		-	-	227.5 (208–265.5)	-	**-**	-	252 (196.5 – 289)	-	0.745
**HbA1c (%)**		0.009	−1 (−1.8 to −0.2)	7.56 ± 1.72	8.68 ± 1.91	**0.001**	−0.5 (−1.75–0.25)	7.52 ± 1.83	8.45 ± 1.92	0.53

*IDD* insulin daily dose; ^a^*P* value was obtained using analysis of covariance (ANCOVA) unless specified (*: independent *t*-test was used; †: chi-square test was applied); ^b^*P* value was obtained from paired-samples *t* tests for parametric variables or Wilcoxon rank-sum test for non-parametric variables. *P* comparison within the group *P*^1^ comparison between the 3 groups

## Discussion

Intensified insulin therapy is currently the mainstay of T1D management in an attempt to achieve tight glycemic control. However, insulin therapy intensification imposes the increased risk of hypoglycemia especially in toddlers and preschool children [22]. Little evidence is available about the effect of different basal insulins on glycemic control and hypoglycemia frequency among toddlers and preschool children with T1D, with only very few trials performed in this age group [6, 22–24].

### Efficacy in terms of glycemic control (HbA1c), CGM, and hospital admission with DKA

In the present study, young children with T1D receiving insulin degludec and insulin glargine showed significantly lower HbA1c 6 months post-therapy than baseline, which was not observed in those who were on NPH. In concordance with these results, a prospective 6‐month study including 14 children with T1D younger than 6 years old who received insulin glargine showed a drop in the average HbA1c without increasing the frequency of severe hypoglycemia [22]. Another two studies comparing insulin glargine to NPH insulin in young children with T1D showed overall improvement in glycemic control in those using insulin glargine compared to NPH insulin [23, 24]. The PRESCHOOL study comparing insulin glargine to NPH insulin in 125 preschool children found a slight increase of the HbA1c in the NPH group with a slight decrease in the insulin glargine group at the study endpoint (after 6 months). However, the mean CGM reading of the insulin glargine group was significantly lower during the day and higher overnight than the NPH group [25].

As for insulin degludec, a study including 9 children younger than 7 years, showed a drop in the HbA1c and a decrease in the frequency of hypoglycemic episodes in children on insulin degludec [26]. Another study comparing insulin degludec to insulin detemir showed a reduction in the rates of hyperglycemia with ketosis with insulin degludec versus insulin detemir; however, this difference did not reach statistical significance [27].

Glycemic variability particularly among young children with T1D has a negative impact on brain growth and volume [28], and endothelial function with an increased risk of complications [7]. However, few studies addressed the time in range and coefficient of variation among toddlers and preschoolers with T1D [7, 29]. In the present study, the mean time in the range of the 30 studied toddlers and preschoolers with T1D was 55.07% and the mean coefficient of variation was 42.82%. In agreement with these results, a study involving 43 preschool children on CSII showed a mean coefficient of variation of 46.1% [7]. Moreover, a recent multicenter study from the USA on 143 children aged 2–8 years with T1D reported a mean time in the range of 40% for young children on multiple daily injection or insulin pump, using masked CGM [29].

Regarding different basal insulins, no significant difference in the mean time in range was found between the insulin degludec and insulin glargine groups but in both, the mean was significantly higher than that of the NPH group. As for the coefficient of variation, it was significantly different between the three groups being lowest in the insulin degludec group than the insulin glargine and the NPH insulin groups. No previous data were found assessing the time in range on insulin degludec, insulin glargine, and NPH insulin in such a young age group. Data from adult studies showed that insulin degludec was superior to insulin glargine in terms of glycemic variability in a multicenter crossover randomized study on 46 adults with T1D [30]. Another open-label randomized control study on 20 adults with T1D showed that treatment with insulin degludec was associated with a lower coefficient of variation without significant difference in hypoglycemia than insulin glargine [31].

### Safety in terms of hypos

In the present study, no significant difference in the frequency of hypoglycemia and severe hypoglycemia was found between the insulin degludec and insulin glargine groups; both being significantly lower than the NPH group. Similarly, Urakami and colleagues noted no significant difference in the frequencies of overall hypoglycemia with insulin degludec and insulin glargine. However, they found a significant decrease in nocturnal hypoglycemia from the baseline with insulin degludec but not with insulin glargine [32]. In agreement with these data, Rollin and colleagues found a significant decrease in the frequency of hypoglycemia and clinically significant hypoglycemia among children with T1D receiving insulin glargine than those receiving NPH insulin [33].

Regarding daily insulin requirements, toddlers and preschoolers receiving insulin degludec and insulin glargine required lower IDD than those receiving NPH insulin. Moreover, toddlers and preschoolers on insulin glargine had significantly lower IDD at the end of the study than at baseline. In agreement with these results, the PRESCHOOL study showed significantly lower mean IDD in young children receiving insulin glargine than those on NPH insulin [24]. Moreover, Predieri and colleagues found no significant difference in the IDD in children and adolescents with T1D on insulin degludec compared to insulin glargine [34].

One limitation of our study is the relatively small sample size and the evaluation of half the study group only using CGMS which could limit the generalizability of the data. This was attributed to the financial cost of the CGMS. Therefore, further larger longitudinal studies are needed to identify the effect of different intermediate, long-acting and ultralong-acting basal insulins on glycemic control and the risk of hypoglycemia among toddlers and preschoolers with T1D.

## Conclusion

The use of insulin degludec and insulin glargine results in better glycemic control with reduced HbA1c and improved time in range, and lower insulin daily dose than NPH insulin among toddlers and preschoolers with T1D. Yet, glycemic variability was highest in the insulin degludec group. Thus, the use of insulin degludec and glargine is recommended in toddlers and preschoolers with T1D owing to their better glycemic control with reduced risk of hypoglycemia and lower IDD than NPH insulin.

## Data Availability

Data will be available upon request.
